# Indikationen und Ergebnisse der Radiojodtherapie beim differenzierten Schilddrüsenkarzinom

**DOI:** 10.1007/s00104-020-01248-x

**Published:** 2020-08-04

**Authors:** B. Riemann

**Affiliations:** Klinik für Nuklearmedizin, Universitätsklinikum Münster, Albert-Schweitzer-Campus 1, Münster, Deutschland

**Keywords:** Risikoprofil, Prognose, Nebenwirkungen, Adjuvante Behandlungsoption, Interdisziplinäres Behandlungskonzept, Risk profile, Prognosis, Side effects, Adjuvant treatment options, Interdisciplinary treatment concept

## Abstract

**Hintergrund:**

Bösartige Schilddrüsentumoren sind die häufigsten bösartigen endokrinen Tumoren, die ungefähr 1 % aller malignen Tumoren umfassen. In den letzten Jahren ist die Inzidenz des differenzierten Schilddrüsenkarzinoms (DTC) deutlich angestiegen, insbesondere der kleinen papillären Karzinome.

**Fragestellung:**

Dargestellt werden Indikationen und Ergebnisse der Radiojodtherapie (RJT) beim DTC.

**Material und Methoden:**

Es erfolgt die Durchsicht der aktuellen Literatur und Leitlinien zur RJT des DTC.

**Ergebnisse:**

Die RJT stellt die wichtigste adjuvante Behandlungsoption des DTC dar und wird generell gut vertragen.

**Schlussfolgerungen:**

Verglichen mit anderen Tumoren hat das DTC durch die Kombination von Operation und RJT eine sehr gute Prognose mit einer durchschnittlichen Überlebensrate von über 90 %.

## Abstract (en)

**Background:**

Malignant tumors of the thyroid gland are the most frequent malignant endocrine neoplasms, comprising approximately 1% of all malignant tumors. In recent years there has been a clear rise in the incidence of differentiated thyroid carcinomas (DTC), especially small papillary carcinomas.

**Objective:**

Indications for and results of radioiodine treatment (RAI) of DTC.

**Material and methods:**

A review of the current literature and guidelines of RAI in DTC was carried out.

**Results:**

The use of RAI is the most important adjuvant treatment option for DTC and is generally well-tolerated.

**Conclusion:**

Due to the combination of surgery and RAI the DTC has a very good prognosis compared with other cancers, with an average survival rate of more than 90%.

Das von den Schilddrüsenhormon bildenden Follikelzellen ausgehende sog. „differenzierte Schilddrüsenkarzinom“ („differentiated thyroid carcinoma“ [DTC]) ist mit etwa 8000 Neudiagnosen pro Jahr in Deutschland der häufigste bösartige endokrine Tumor. Das DTC hat durch die Kombination von Operation und Radiojodtherapie (RJT) eine exzellente Prognose mit 5‑Jahres-Überlebensquoten von über 97 % im Niedrigrisikokollektiv und 83 % bei Patienten mit primär organüberschreitendem Wachstum oder Fernmetastasen [1].

## Prinzip und Indikationen der Radiojodtherapie

Bei der Therapie benigner und maligner Schilddrüsenerkrankungen mit ^131^Iod wird die Tatsache genutzt, dass es ebenso wie stabiles Iod aus der Nahrung sehr effizient mithilfe des humanen Natrium-Iodid-Symporters (hNIS) aus der Blutbahn von den Follikelepithelzellen der Schilddrüse und zumeist auch von Schilddrüsentumorgewebe aufgenommen und mit einer effektiven Halbwertszeit von bis zu mehreren Tagen gespeichert wird. Die beim radioaktiven Zerfall von ^131^Iod ausgehende Betastrahlung mit einer maximalen Reichweite von 2 mm – Mittel 0,4 mm – im Gewebe ist für 95 % des therapeutischen Effekts am Schilddrüsen(tumor)gewebe verantwortlich. Aufgrund der kurzen Reichweite der Betastrahlung ist die Strahlenexposition des übrigen Körpers gering. Therapeutische Effekte sind Apoptose und Nekrose von Thyreozyten und/oder Schilddrüsenkarzinomzellen. Die Wirkung tritt innerhalb von 6 bis 12 Monaten ein. Die vom ^131^Iod ebenfalls emittierte Gammastrahlung wird für die Dosimetrie und die diagnostische Ganzkörperszintigraphie genutzt.

Eine regelhafte Indikation zur adjuvanten RJT besteht im Falle aller DTC. Ausnahmen sind das papilläre Mikrokarzinom (PTMC), der nichtinvasive follikuläre Tumor mit Kernmerkmalen des papillären Schilddrüsenkarzinoms (NIFTP) und das minimal-invasive follikuläre Schilddrüsenkarzinom ohne Angioinvasion. Hier wird im Hinblick auf das Active-surveillance-Konzept die Indikation zur RJT wieder verstärkt kontrovers diskutiert. So kann beim PTMC mit Risikofaktoren wie Multifokalität, Organkapselinfiltration, gering differenziertem Subtyp, infiltrativem Tumorwachstum, desmoplastischer Fibrose, ggf. BRAF V600E-Mutation, Tumordurchmesser 6–10 mm, nicht inzidentellem Karzinomnachweis, Familiarität oder perkutaner Vorbestrahlung der Halsweichteile eine RJT erfolgen [2]. Metastasierte DTC stellen eine Indikation zur hoch dosierten RJT dar, sofern diese ausreichend Iod anreichern und keine Möglichkeit zu einem chirurgischen Vorgehen besteht.

## Durchführung und Ergebnisse

Voraussetzung für die Durchführung einer Therapie und/oder Diagnostik mit ^131^Iod ist eine ausreichende TSH(Thyreoidea-stimulierendes Hormon)-Stimulation (günstig TSH >30 µU/ml), die exogen durch Gabe von gentechnisch hergestelltem rekombinantem TSH bei laufender L‑Thyroxin-Medikation oder endogen durch eine L‑Thyroxin-Karenz erzielt wird [2]. Weiterhin gelten die Vermeidung bzw. ggf. der Ausschluss einer Kontamination mit stabilem Iod durch die Nahrung (Seefisch, Algenpräparate) oder durch die Gabe jodhaltiger Röntgenkontrastmittel (z. B. Computertomographie [CT]/Angiographie) sowie der Ausschluss einer Schwangerschaft, die eine absolute Kontraindikation darstellt.

In Deutschland muss die RJT gemäß der Richtlinie Strahlenschutz in der Medizin unter stationären Bedingungen durchgeführt werden. Der Aufenthalt der Patienten im Kontrollbereich dauert üblicherweise einige Tage. Die adjuvante RJT erfolgt durch Gabe von in der Regel 2–3,7 GBq ^131^I (^131^Iod). Im Regelfall wird die Aktivität oral in Kapselform nach einer Nahrungskarenz von 4 h vor und 1 h nach Einnahme verabreicht. Im Fall einer endogenen TSH-Stimulation wird 48 h nach Applikation der ^131^I‑Aktivität mit der Einnahme von L‑Thyroxin begonnen. Während des stationären Aufenthalts wird der zeitliche Aktivitätsverlauf im Patienten gemessen. Der genaue Zeitpunkt der posttherapeutischen ^131^I‑Ganzkörperszintigraphie (meist nach 72 h) richtet sich nach der verbliebenen Restaktivität und den Abbildungseigenschaften der Gammakamera und der SPECT(„single photon emission computed tomography“)(/CT) [2].

In Deutschland muss die RJT unter stationären Bedingungen durchgeführt werden

Ziele der adjuvanten RJT sind die Optimierung der Nachsorge und Kontrolle des Tumors über die Bestimmung des hochsensitiven Thyreoglobulin (hTg) für die Rezidivdiagnostik sowie ein Staging mittels posttherapeutischer ^131^I‑Ganzkörperszintigraphie. Der Nutzen der adjuvanten RJT wird in einer Reihe von Studien belegt. So zeigten Maier et al. bei DTC-Patienten älter als 60 Jahre, die eine adjuvante RJT zusätzlich zur Operation erhielten, paradoxerweise eine signifikante Verlängerung ihrer Lebenserwartung im Vergleich zur Alters‑, Geschlechts- und Kalenderjahr-abgeglichenen Normalbevölkerung in Deutschland [1].

Acht Wochen nach jeder ^131^I‑Therapie ist eine Kontrolle von Blutbild, basalem TSH zur Beurteilung der Effektivität der L‑Thyroxin-Medikation und des Tumormarkers Thyreoglobulin (Tg) obligatorisch. Die ^131^I‑Ganzkörperszintigraphie zur Kontrolle des Therapieerfolgs wird üblicherweise 6 bis 12 Monate nach vorangegangener Therapie durchgeführt; bei niedriger Wahrscheinlichkeit für iodavide Metastasen sollte eine exogene TSH-Stimulation bevorzugt werden [3]. Nach intramuskulärer Gabe von jeweils 0,9 mg rh(rekombinantes humanes)TSH an Tag 1 und 2 folgen die Applikation von 150–370 MBq ^131^I an Tag 3 sowie die hTg-Bestimmung und ^131^I‑Szintigraphie an Tag 5. Bei erfolgreicher RJT sollten kein ^131^I‑Uptake mehr nachweisbar und das stimulierte Serum-hTg auf unter 1 ng/dl gesenkt sein.

Die RJT ist darüber hinaus eine sehr wirkungsvolle Therapieoption bei jodspeichernden Fernmetastasen, für die keine Option zur operativen Therapie besteht. Hierbei werden Standardaktivitäten zwischen 4 und 11 GBq oder individuelle Aktivitätsabschätzungen mittels dosimetrischer Verfahren (Blutdosis/rotes Knochenmark, Tumordosis) eingesetzt. Bislang liegen jedoch noch keine prospektiven, randomisierten Dosimetrie-Therapie-Optimierungsversuche vor [4]. Zum Schutz von Speicheldrüsen und Magen, die ebenfalls Iod anreichern, können die Patienten Sialagoga (z. B. Zitronensaft 24 h nach Applikation) und Protonenpumpenblocker erhalten. Zur Reduktion der Strahlenexposition der Blase sollte reichlich Flüssigkeit zugeführt werden und die Blase insbesondere zu Beginn der Therapie regelmäßig entleert werden. Jede therapeutische Applikation von ^131^I wird von einer posttherapeutischen ^131^I‑Ganzkörperszintigraphie zum spezifischen Nachweis und/oder Ausschluss jodspeichernden Tumorgewebes gefolgt.

**Figure 1:**
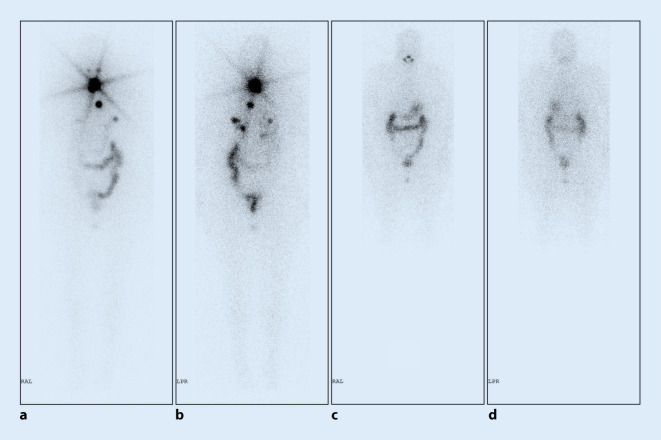

Jodspeichernde Lungenmetastasen sind einer hoch dosierten ^131^I‑Therapie sehr gut zugänglich (Abb. 1). So ist eine Kuration der im jungen Alter häufigen disseminierten pulmonalen Filialisierung bei papillärem DTC, die zumeist nur in der ^131^I‑Ganzkörperszintigraphie erkennbar ist, mit 5‑Jahres-Überlebensquoten über 90 % möglich. Dies liegt insbesondere an einer höheren Expression des hNIS bei Kindern und Jugendlichen. Daher ist ihr Gesamtüberleben exzellent. Allerdings müssen bei ihnen mögliche Späteffekte der RJT wie die Lungenfibrose beachtet werden. Aus diesem Grund sollte die RJT zurückhaltender als bei Erwachsenen eingesetzt und keine komplette Remission angestrebt werden [5]. Die Indikation muss im interdisziplinären Tumorboard gestellt werden, und die Behandlung sollte nur in Referenzzentren erfolgen.

Jodspeichernde Knochenmetastasen lassen sich mit wiederholten ^131^I‑Therapien in Einzelfällen heilen und in den allermeisten Fällen langfristig in ihrer Progression hemmen. Nicht jodspeichernde Tumormanifestationen können perkutan mit palliativer Intention bestrahlt werden.

## Nebenwirkungen

Eine klinisch symptomatische Strahlenthyreoiditis insbesondere nach subtotaler Schilddrüsenoperation ist durch lokale Kühlung gut zu beherrschen und erfordert nur selten die systemische Gabe von Antiphlogistika. Bei Hirn- und Wirbelsäulenmetastasen sowie großen paratrachealen Tumormassen mit Gefahr kompressionsbedingter Komplikationen wird die Gabe von Glukokortikoiden empfohlen [2]. Bei diesen Patienten müssen die Risiken einer endogenen (Tumorschwellung unter L‑Thyroxin-Karenz) gegenüber einer exogenen TSH-Stimulation abgewogen werden. Unter den chronischen Nebenwirkungen steht die Xerostomie durch radiogene Schädigung der Speicheldrüsen, besonders der Ohrspeicheldrüsen (10–20 %), im Vordergrund. Die anderen Langzeitnebenwirkungen treten zumeist nur bei kumulativen Radiojodaktivitäten über 22 GBq auf. Selten sind permanente Knochenmarkdepression, Leukämie in ca. 1 % bei diesen Aktivitäten, Lungenfibrose selten bei intensiv jodspeichernden Lungenmetastasen, Azoospermie bei Erwachsenen. Ein Anstieg der Fehlgeburten- oder Missbildungsrate bei Nachkommen wurde bisher nicht beobachtet.

## Nachsorge

Beim DTC wird das Konzept einer lebenslangen Nachsorge vertreten. Ziel der Nachsorge ist neben der optimalen Durchführung der L‑Thyroxin-Medikation die Erkennung lokoregionärer Rezidive und/oder Fernmetastasen vor Einsetzen einer klinischen Symptomatik. Bei unauffälligem Verlauf werden ambulante Nachsorgen in 1‑ bis 2‑jährigen Abständen durchgeführt. Während eine hTg-Bestimmung mit einem hochsensitiven Assay unter TSH-Suppression 90 % aller Patienten mit Metastasierung identifiziert, werden bei einem Cut-off von 2 ng/dl durch eine hTg-Bestimmung unter TSH-Stimulation nahezu 100 % der Patienten mit Metastasierung eines DTC erfasst [6].

Beim DTC wird das Konzept einer lebenslangen Nachsorge vertreten

Die Konstellation einer Erhöhung des hTg ohne korrespondierenden ^131^I‑Uptake, das TENIS(„thyroglobulin elevated and negative iodine scintigraphy“)-Syndrom, erfordert eine weiterführende Diagnostik. Tumorrezidive und Metastasen mit verminderter hNIS-Expression können durch einen gesteigerten ^18^F‑FDG(Fluordesoxyglucose)-Metabolismus in der PET(Positronenemissionstomographie)-CT nachgewiesen werden [7]. Derzeit wird kontrovers diskutiert, ob Patienten mit einem fehlenden ^131^I‑Uptake in der Szintigraphie als Radiojod-refraktär bezeichnet werden sollten [8]. Dies liegt daran, dass es bislang noch keine allgemein anerkannte Definition des Begriffs Radiojod-refraktär gibt. Auch wenn bei Patienten mit einem fehlenden ^131^I‑Uptake in der Szintigraphie von einer geringeren Wahrscheinlichkeit eines guten und andauernden Ansprechens auf die RJT auszugehen ist, bedeutet dies nicht, dass sie nicht mehr davon profitieren können. Insbesondere vor dem Hintergrund sehr eingeschränkter Therapieoptionen sollte in diesen komplexen Fällen individuell zusammen mit den Patienten evaluiert werden, ob eine RJT einen potenziellen Benefit bedeuten kann.

## Fazit für die Praxis

- Die Radiojodtherapie (RJT) stellt die wichtigste adjuvante Behandlungsoption des differenzierten Schilddrüsenkarzinoms (DTC) dar.
- Ziele der am individuellen Risikoprofil ausgerichteten RJT sind die Behandlung des metastasierten DTC und die Optimierung der Nachsorge.
- Die RJT wird generell gut vertragen.
- Aufgrund des interdisziplinären Behandlungskonzepts mittels Chirurgie und RJT ist die Prognose des DTC sehr gut.
